# Integrated bioinformatics analysis and screening of hub genes in papillary thyroid carcinoma

**DOI:** 10.1371/journal.pone.0251962

**Published:** 2021-06-11

**Authors:** Rong Fan, Lijin Dong, Ping Li, Xiaoming Wang, Xuewei Chen

**Affiliations:** 1 Central Laboratory, Tianjin Xiqing Hospital, Tianjin, PR China; 2 Editorial Department of Education and Research Security Centre, Logistic University of Chinese People’s Armed Police Force, Tianjin, PR China; 3 Southwest Medical University, Luzhou City, Sichuan Province, PR China; 4 Department of Operational Medicine, Tianjin Institute of Environmental and Operational Medicine, Tianjin, PR China; University of Salemo, ITALY

## Abstract

**Background:**

With the increasing incidence of papillary thyroid carcinoma (PTC), PTC continues to garner attention worldwide; however its pathogenesis remains to be elucidated. The purpose of this study was to explore key biomarkers and potential new therapeutic targets for, PTC.

**Methods:**

GEO2R and Venn online software were used for screening of differentially expressed genes. Hub genes were screened via STRING and Cytoscape, followed by Gene Ontology and KEGG enrichment analysis. Finally, survival analysis and expression validation were performed using the UALCAN online software and immunohistochemistry.

**Results:**

We identified 334 consistently differentially expressed genes (DEGs) comprising 136 upregulated and 198 downregulated genes. Gene Ontology enrichment analysis results suggested that the DEGs were mainly enriched in cancer-related pathways and functions. PPI network visualization was performed and 17 upregulated and 13 downregulated DEGs were selected. Finally, the expression verification and overall survival analysis conducted using the Gene Expression Profiling Interactive Analysis Tool (GEPIA) and UALCAN showed that LPAR5, TFPI, and ENTPD1 were associated with the development of PTC and the prognosis of PTC patients, and the expression of LPAR5, TFPI and ENTPD1 was verified using a tissue chip.

**Conclusions:**

In summary, the hub genes and pathways identified in the present study not only provide information for the development of new biomarkers for PTC but will also be useful for elucidation of the pathogenesis of PTC.

## Introduction

Over the past few years, thyroid diseases have garnered increasing attention and the incidence of thyroid cancer has markedly increased [1]. Thyroid cancer is divided into the following five types according to histogenesis and morphology: anaplastic, Hurthle cell, follicular, medullary, and papillary thyroid carcinoma, of which papillary thyroid carcinoma or papillary thyroid cancer (PTC) accounts for 80% of the overall incidence [2]. With the widespread use of neck ultrasound and fine-needle aspiration biopsy for diagnosis, the number of deaths attributable to thyroid cancer has significantly reduced [3]. However, the survival rate is affected by several factors, and the prognosis of PTC remains extremely poor despite the adoption of multiple treatment strategies such as thyroidectomy, radioiodine treatment, and chemotherapy [4]. Therefore, the early prevention and diagnosis of thyroid cancer remains a necessity and concern for doctors and scientists, and exploration of potential key biomarkers and novel therapeutic targets in PTC is imperative for doctors and patients alike.

With the large-scale application of high-throughput screening technology, we have identified several novel genes associated with disease initiation and progression and have achieved a comprehensive understanding of the molecular mechanisms of development of various tumors [5–8]. In the present study, we used bioinformatics methods to mine microarray data from the Gene Expression Omnibus (GEO) database, and analyzed the DEGs between PTC and normal tissues. Thereafter, we constructed a protein-protein interaction (PPI) network, performed functional enrichment and survival analysis, identified three hub genes, and discovered the biological processes and signaling pathways associated with PTC development. In conclusion, the integrated analysis provided insights for the development of new biomarkers for PTC, which may be valuable for conducting further research into the mechanisms of PTC as well as for use in clinical applications in diagnosis, prognosis, and therapy.

## Materials and methods

### Acquisition of microarray data

We obtained high-throughput gene expression profiles of PTC and normal thyroid tissues from the GEO database. The independent datasets GSE3678, GSE33630, and GSE53157 were selected, and all were based on the GPL570 platforms, including 7 PTC tissues and 7 normal tissues, 49 PTC tissues, 45 normal tissues, 7 PTC tissues, and 3 normal tissues.

### Screening of DEGs

The DEGs between PTC and normal tissues were screened using the GEO2R tool. GEO2R was used to compare and identify DEGs present in two or more sets of data in the GEO series. We used the adjusted *P*-value instead of the non-adjusted *P*-value to restrict the false-positive rate. The cut-off criteria of |logFC| ≥1 and adjusted *P*-value < of 0.05 were considered statistically significant. The DEGs in the three datasets were screened using the Venn software online. DEGs with logFC≥1 were considered as upregulated genes, whereas DEGs with logFC<-1 were considered as downregulated genes.

### Enrichment analysis via GO and KEGG pathway

To characterize the functional roles of the DEGs, we used the Database for Annotation, Visualization, and Integrated Discovery (DAVID, version 6.8) [9] for GO enrichment analysis, which included biological process (BP), cellular component (CC), molecular function (MF), and KEGG pathway analysis with a cut-off *P*-value of <0.05. Visualization of the enrichment analysis was performed using the online platform Image GP (http://www.ehbio.com/ImageGP/index.php/Home/Index/index.html).

### Construction of the PPI network and analysis of the module

The PPI network was constructed using the Search Tool for the Retrieval of Interacting Genes/Proteins (STRING) database to reveal the relationships of DEGs based on a minimum required interaction score = 0.4 [10]. The PPI network was illustrated and analyzed using the Cytoscape (version 3.6.1) software [11]. Additionally, the core modules of the PPI network were screened using MCODE (node score cutoff = 0.2, degree cutoff = 2, k-score = 2, and maximum depth = 100).

### Survival analysis and validation of hub gene expression

UALCAN was used to analyze the relationship between key gene expression and survival of patients with PTC, which is an established resource for analyzing transcriptome data of cancers based on The Cancer Genome Atlas (TCGA) [12]. The Gene Expression Profiling Interactive Analysis tool (GEPIA) [13] was used to analyze RNA expression data based on thousands of samples from TCGA and GTEx projects. Statistical significance was set at *P* < 0.05.

### Tissue samples and immunohistochemistry (IHC)

Human papillary thyroid cancer tissue microarray sections (HThyP120CS02) were obtained from Shanghai Outdo Biotech Co. Ltd. (Shanghai, China). The tissue samples were procured from 62 patients with papillary thyroid cancer, which consisted of 58 cancer tissues, 58 para-cancerous tissues, 3 normal thyroid tissues, and 1 chronic lymphocytic thyroiditis tissue. The two-step EnVision method was used to perform immunohistochemical experiments, along with the use of different primary antibodies against LPAR5 (1:50). The study was conducted with the consent of human subjects and approved by the ethics committee of Tianjin Xiqing Hospital and the Shanghai Outdo Biotech Company (Shanghai, China). All experiments were conducted in accordance with the Declaration of Helsinki (1964). The slides were analyzed using the Image-Pro PLUS software program (Media Cybernetics, Inc. USA).

### Statistical analysis

Statistical analysis was performed using SPSS 22.0 and GraphPad Prism 8.0. Results are presented as mean ± standard deviation. The Student’s *t*-test was used to compare differences between two sample groups. Statistically significant changes are indicated with asterisks, where *, **, and *** represent *P* < 0.05, *P* < 0.01, and *P* < 0.001, respectively.

## Results

### Identification of DEGs in papillary thyroid cancers

**Fig 1:**
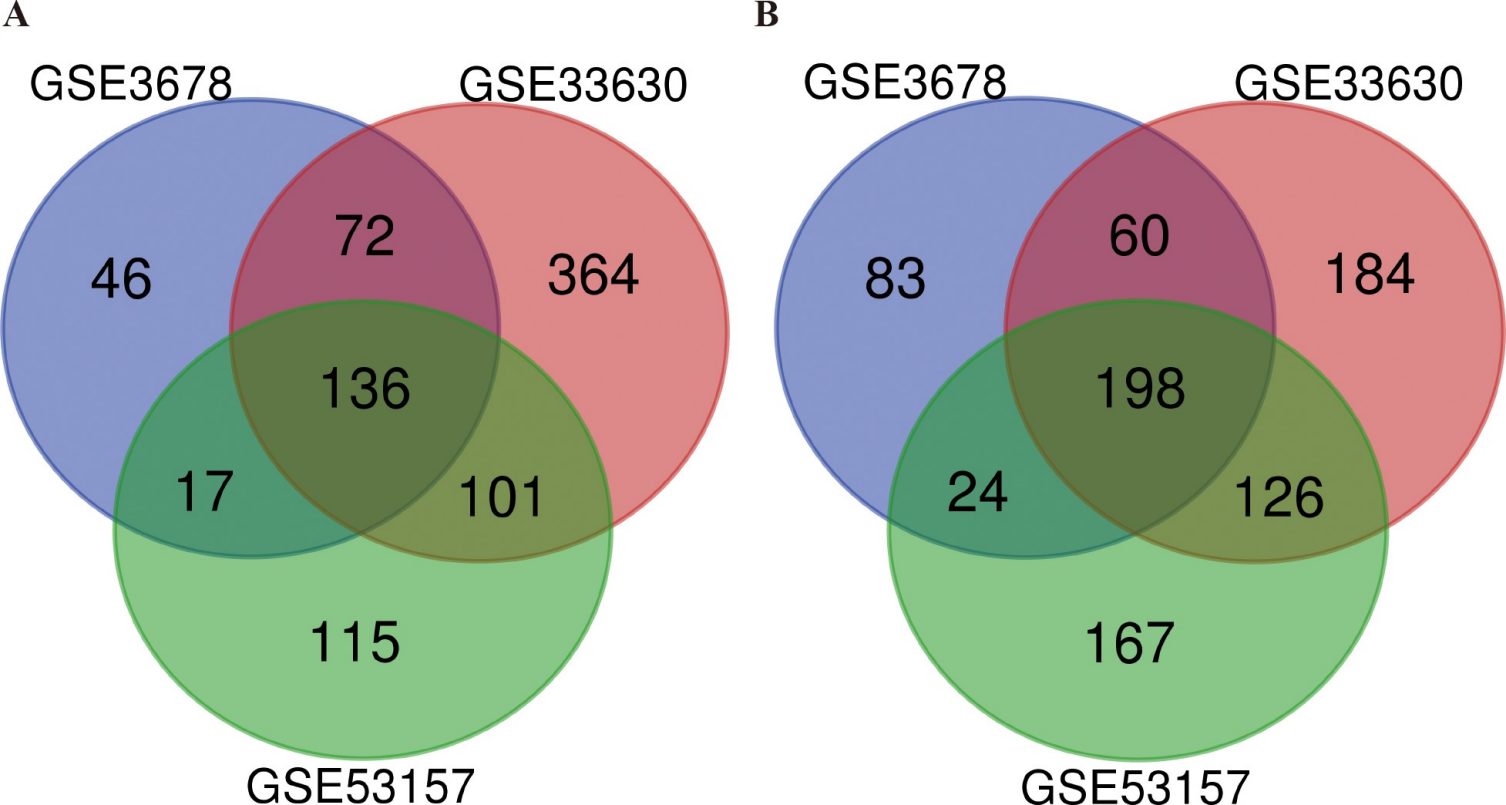
Selection of 334 common DEGs from the three datasets (GSE3678, GSE33630, and GSE53157). A. Venn diagram illustrating 136 upregulated DEGs (logFC≥1); B. Venn diagram illustrating 198 downregulated DEGs (logFC<1). The number denotes the number of genes shared between/among subtypes or the number of unique genes.

In this study, we selected and downloaded three GEO datasets and extracted DEG data based on the cut-off criteria. Data on a total of 63 papillary thyroid cancers and 55 normal thyroid tissues were obtained in this study. There were 636 DEGs in GSE3678, including 271 upregulated and 365 downregulated genes, 1241 DEGs in GSE33630, including 673 upregulated and 568 downregulated genes, and 884 DEGs in GSE53157, including 369 upregulated and 515 downregulated genes. Using the Venn diagram software, we detected a total of 334 common DEGs, which included 136 upregulated genes and 198 downregulated genes in PTC tissues (Table 1 and Fig 1).

**Table 1:** Screening of 334 commonly differentially expressed genes from three profile datasets, including 136 upregulated and 198 downregulated genes in PTC tissues, compared to normal thyroid tissues.

DEGs	Gene symbol
**Upregulated**	CDH3, KLHDC8A, KCNJ2, GOLT1A, TNIK, PDZK1IP1, BID, RXRG, MPZL2, GDF15, FRMD3, DOCK9, MET, PTPRE, FAM20A, IGSF3, ALOX15B, ALOX5, EPS8, NGEF, ITGA3, DUSP6, PRSS2, CD55, NOD1, CFI, DDB2, DPP4, NPC2, DUSP4, CORO2A, ATP11A, ST8SIA4, LGALS3, PDLIM4, PLAG1, SYT12, RASD2, NFE2L3, TTC39B, DUSP5, LPAR5, TUSC3, FAXC, MYEF2, PDE5A, TACSTD2, PRR15, CDC42EP3, SNX25, RYR1, SPINT1, LRRK2, LOC101927705///P4HA2, NRP2, PRSS23, SIPA1L2, CHI3L1, CITED1, C19orf33, NR2F1-AS1, SERPINA1, TGFA, PLXNC1, LIPH, GGCT, LONRF2, TIAM1, FN1, SYTL1, RUNX2, SNX22, C4orf48, SYTL5, SERINC2, KRT19, ZCCHC12, LAMB3, LMO3, TENM1, SHROOM4, EMILIN2, ENTPD1, GALE, SDC4, METTL7B, HEY2, ETV5, ABCC3, NRCAM, LOC101928269///LOC100506403///RUNX1, GABRB2, SLC35F2, TNRC6C-AS1, TMPRSS4, SLC34A2, BICD1, STK32A, MYH10, RAB27A, TBC1D2, LRP4, CLDN1, LOC729461///FAM230B///FAM230C, TGFBR1, GABBR2, CCND1, NHSL2, MEGF9, CDH6, GALNT7, SREBF1, PTP4A3, TMC6, TPD52L1, PROS1, CAMK2N1, QPCT, SCEL, MCTP2, ETV1, KCNN4, KDELR3, PCSK2, COL8A1, ZMAT3, CYP1B1, HMGA2, LOC101928195///LOC100996643///MTHFD1L, LOC100507165, PSD3, AGR2, DTX4, CTSC, ARMCX3, XPR1
**Downregulated**	HBA2///HBA1, PKNOX2, MUM1L1, COL9A3, PBX4, IRS4, FGL2, MPPED2, DPT, TBC1D4, CITED2, ASXL3, MFAP4, FOXP2, FAM234B, DLG2, LAYN, TNS3, STXBP5L, SCARA5, TCEAL2, GPR83, RGS16, HGD, PAPSS2, GLT8D2, ID4, DGKI, EML1, GJB6, CDH16, ANO5, GHR, GNAI1, SCUBE3, LIFR, GRAMD2, MAGI2, CSGALNACT1, LYVE1, LOC101930400///AKR1C2, TMPRSS3, SOD3, GDF10, TFCP2L1, OTOS, EML6, STARD13, BCL2, KIAA1324, CXCL12, IPCEF1, RAP1GAP, PLEKHG4B, 2-Mar, AVPR1A, WDR72, SPX, IRS1, TSPAN7, PID1, SMAD9, BTBD11, C4orf47, ELMO1, NCAM1, DIO2, TPO, CASZ1, SORD, PQLC2L, SLC25A33, FCGBP, TBC1D8///RPL31, RYR2, PLA2R1, PKHD1L1, KCNIP4, GSTM3, ESRRG, CHCHD10, HBB, SERTM1, FHL1, RHOJ, SORBS2, MAFB, IP6K3, TFPI, C11orf74, RPS6KA6, MT1G, TMEM171, PKIA, PEG3, CCL21, ST7-AS1, SCN3A, CAPSL, PPARGC1A, DEPTOR, FBLN7, ERO1B, CRABP1, ZFPM2, RNF157-AS1, FHDC1, GNA14, MT1F, SHANK2, ANKS1B, BMP8A, COL23A1, TGFBR3, ADH1B, AKR1C1, ANK2, NUAK2///AKIP1, KIZ, VLDLR, LMOD1, PRKCQ, DIO1, CCDC146, FGFR2, KIT, FABP4, ZDHHC11B///ZDHHC11, FAM167A, FAXDC2, ACACB, LOC440934, LOC646736, CYP7B1, RGS8, PGM5, MIR4683///FZD8, AADACP1, RNF150, AGPAT4, ZMAT4, TBX22, SLC16A2, TPPP, LOC101927137///KIAA1456, CTH, RASSF9, DDX25, TLE4, AKR1C3, SH3GL2, LOC101929480, DIRAS2, AGTR1, FMOD, ADAM22, LOC101060817///GCSH, DPY19L2, CLMN, SNCA, LRIG1, ANKRD18A, AIF1L, L3MBTL4, ADGRV1, RELN, CWH43, IGFBPL1, LOC100506558///MATN2, SLC26A7, WWOX, WASF3, UGT8, CUX2, SOX9-AS1, SLC4A4, TMEM178A, OGDHL, ADGRA3, DYNLRB2, TFF3, AOX1, GPM6A, CLCNKB, MDH1B, SAMD5, CFD, DPP6, TRIM58, INAFM2, FLRT1, MRO, TTC30A, EFEMP1, WFS1, SMOC2, ANKRD37

### GO and pathway enrichment analysis

**Fig 2:**
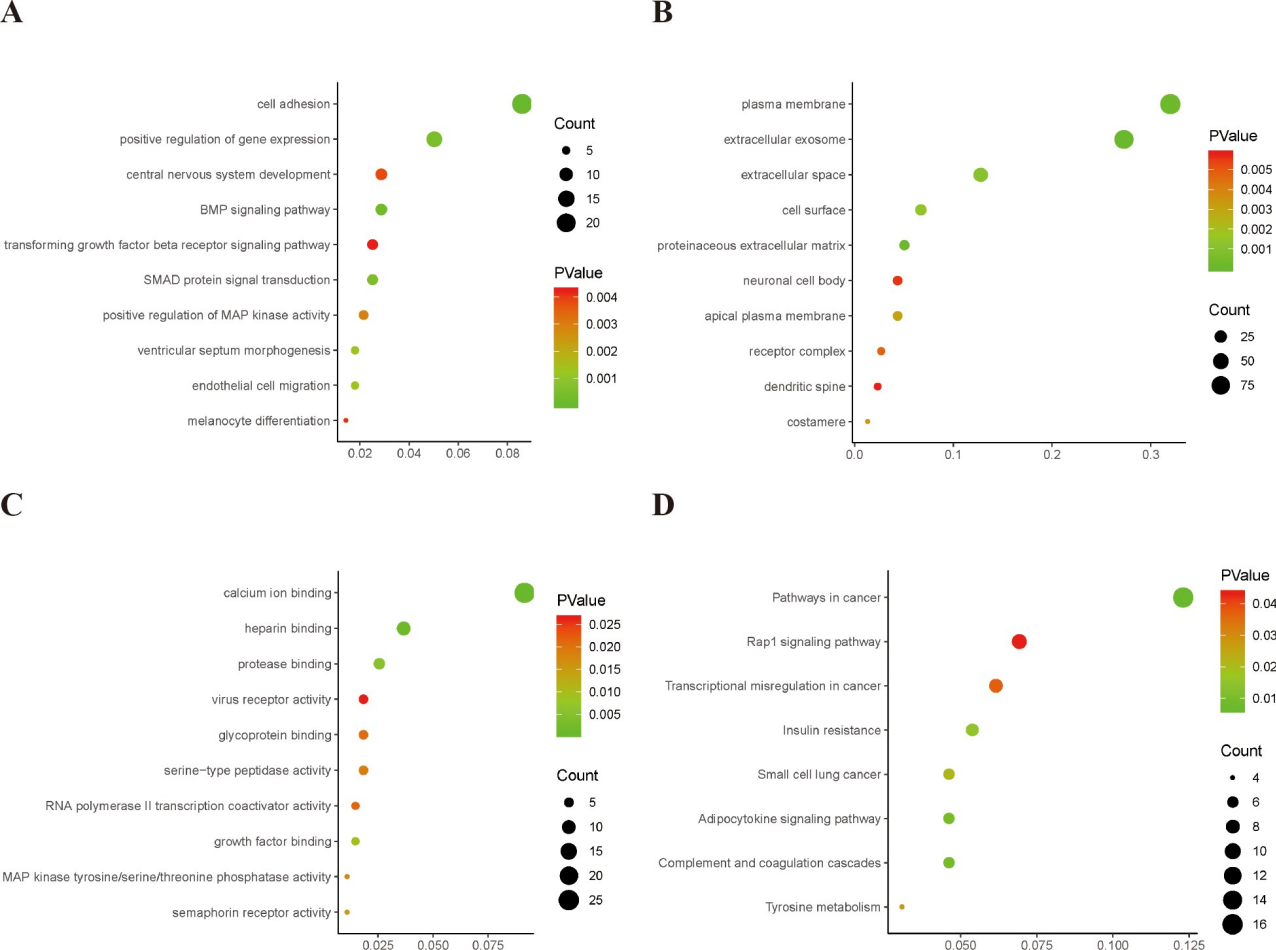
The top 10 gene ontology (GO) and significantly enriched KEGG pathways. A. BP; B. CC; C. MF; D. KEGG pathways. The X axis represents the enrichment levels; the Y axis represents the top 10 remarkably enriched categories. The color of the dot denotes the different *P-value*, and the size of the dot indicates the number of the candidate genes enriched in the GO and KEGG pathways.

DAVID was used for gene ontology functional annotation and biological pathway enrichment analysis of DEGs as a tool for the analysis of genes and proteins. We analyzed upregulated and downregulated genes using the DAVID software and identified 125 significant enrichment terms, including biological processes (BP, 79), molecular functions (MF, 22), and cellular components (CC, 24). As a common method adopted for defining genes and their corresponding RNA or protein products, GO analysis is usually performed to identify the unique biological characteristics of high-throughput transcriptomic or genomic data. BP, CC, and MF are the three main categories in the GO function annotation. In case of BP, DEGs were significantly enriched in cell adhesion, positive regulation of gene expression, BMP signaling pathway, transforming growth factor beta receptor signaling pathway, SMAD protein signal transduction, positive regulation of MAP kinase activity, ventricular septum morphogenesis, endothelial cell migration, and melanocyte differentiation. In case of MF, DEGs were significantly enriched in calcium ion binding, protease binding, glycoprotein binding, serine-type peptidase activity, RNA polymerase II transcription coactivator activity, growth factor binding, MAP kinase tyrosine/serine/threonine phosphatase activity, and semaphorin receptor activity. In case of CC, DEGs were remarkably enriched in the plasma membrane, extracellular space, cell surface, proteinaceous extracellular matrix, neuronal cell body, apical plasma membrane, receptor complex, dendritic spine, and costamere. The top 10 GO terms are shown in Fig 2. Furthermore, KEGG is a collection of databases encompassing data of genomes, drugs, pathways, diseases, and chemical substances. KEGG pathway analysis showed that the DEGs were enriched in eight pathways, including pathways involved broadly in cancer, the Rap1 signaling pathway, transcriptional misregulation in cancer, insulin resistance, small cell lung cancer, adipocytokine signaling pathway, complement and coagulation cascades, and tyrosine metabolism (Fig 2).

### Analysis of the PPI network and module

**Fig 3:**
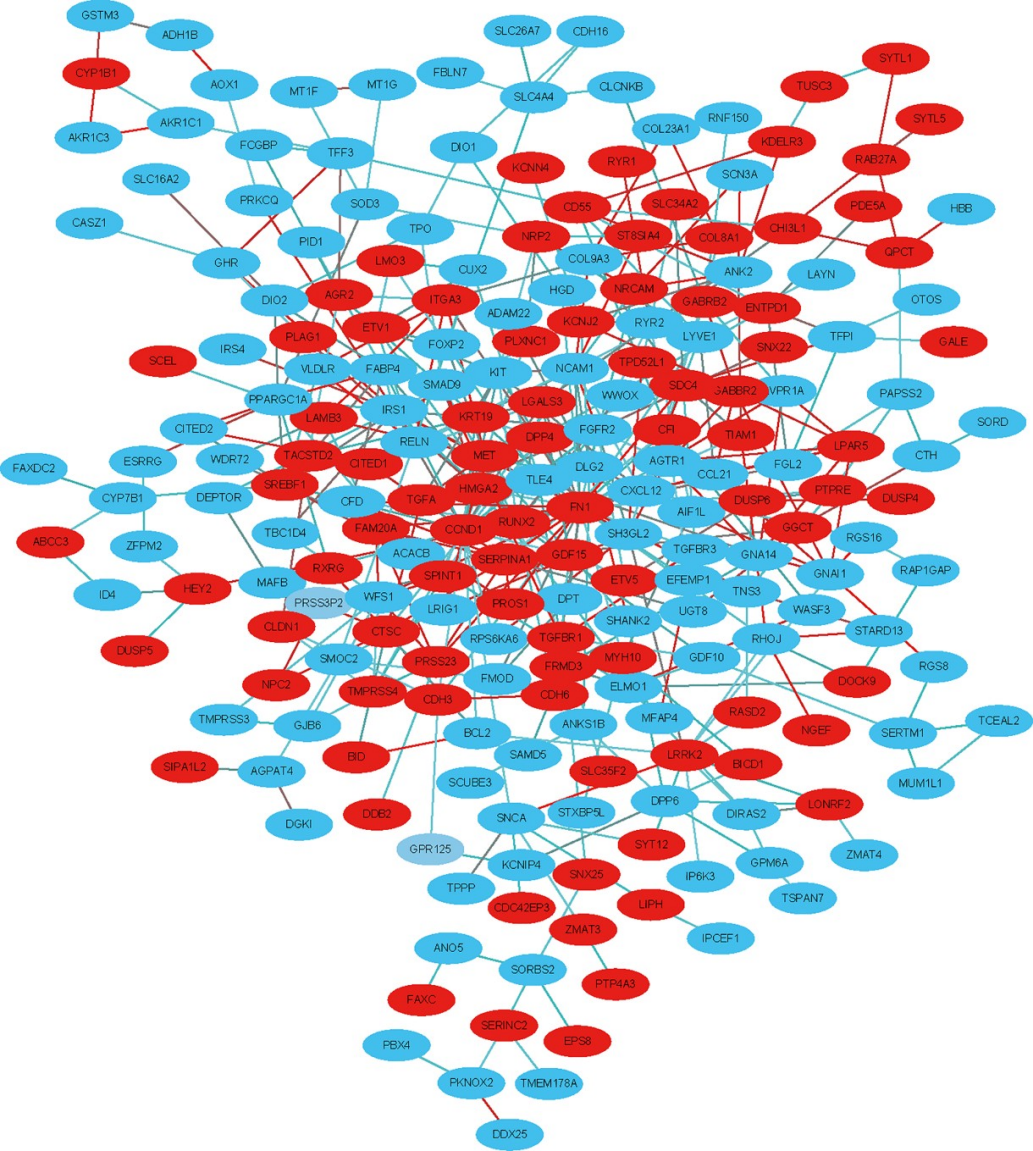
PPI network construction using STRING and Cytoscape. Each node indicates a protein module; the edges represent protein interactions; red circles denote upregulated DEGs and blue circles represent downregulated DEGs.

**Fig 4:**
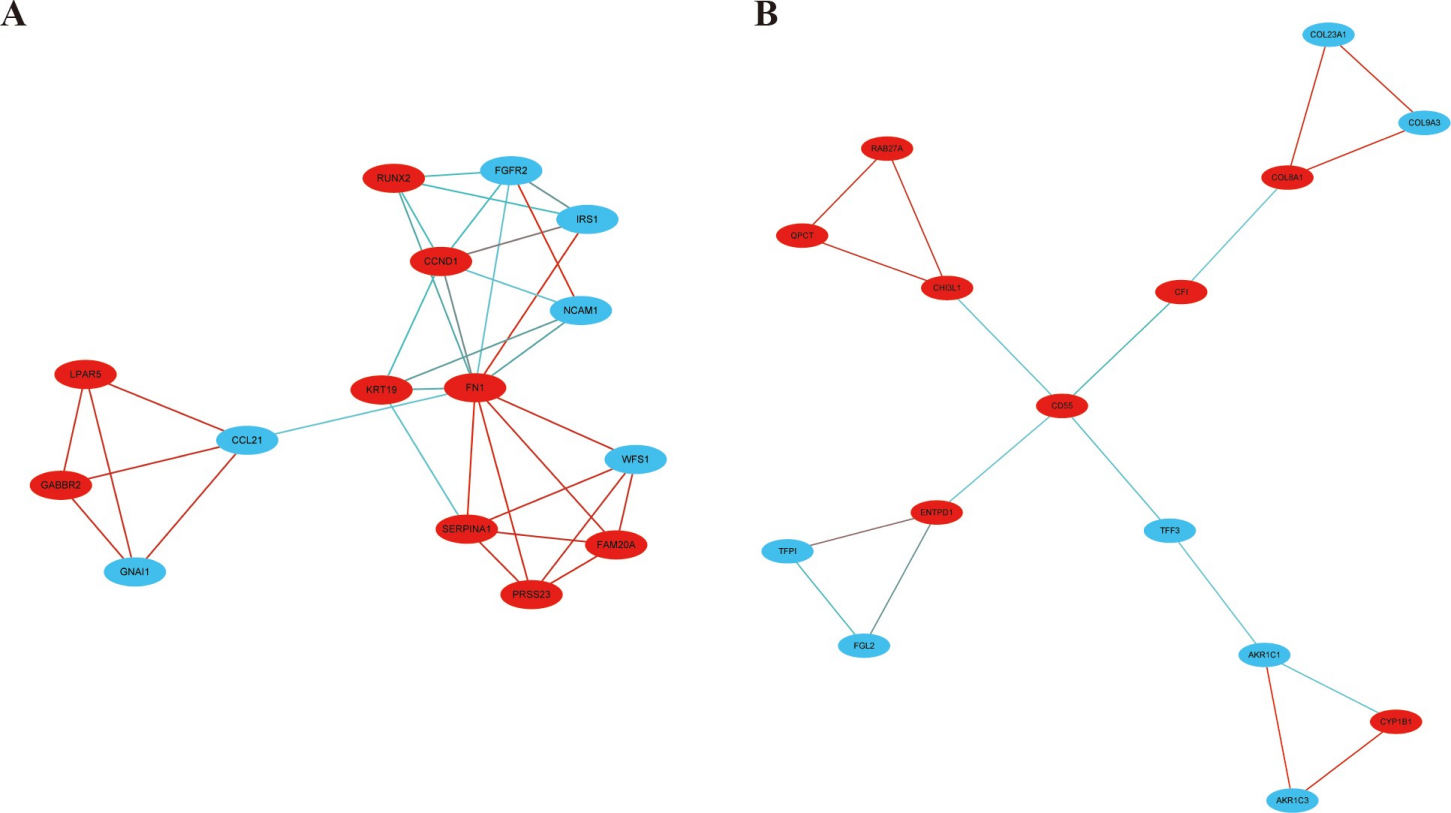
Module analysis by MCODE using Cytoscape. A. module1; B. module2. Red circles represent upregulated DEGs and blue circles denote downregulated DEGs.

Based on STRING and Cytoscape analysis, we constructed a PPI network complex with 241 nodes and 442 edges, including 101 upregulated and 137 downregulated genes. We then performed further analysis by applying Cytoscape MCODE plus and obtained 30 central nodes, including 17 upregulated genes and 13 downregulated genes (Figs 3 and 4, and Table 2).

**Table 2:** Selection of 30 central genes from PPI network, including 17 upregulated and 13 downregulated genes, by using the STRING and Cytoscape software.

DEGs	Gene symbol
**Upregulated**	CCND1, FAM20A, FN1, PRSS23, LPAR5, SERPINA1, KRT19, GABBR2, RUNX2, CHI3L1, CFI, QPCT, CD55, CYP1B1, RAB27A, ENTPD1, COL8A1
**Downregulated**	GNAI1, NCAM1, CCL21, IRS1, WFS1, FGFR2, FGL2, AKR1C3, AKR1C1, TFF3, COL23A1, TFPI, COL9A3

### Selection of hub genes and validation of their expression level

**Fig 5:**
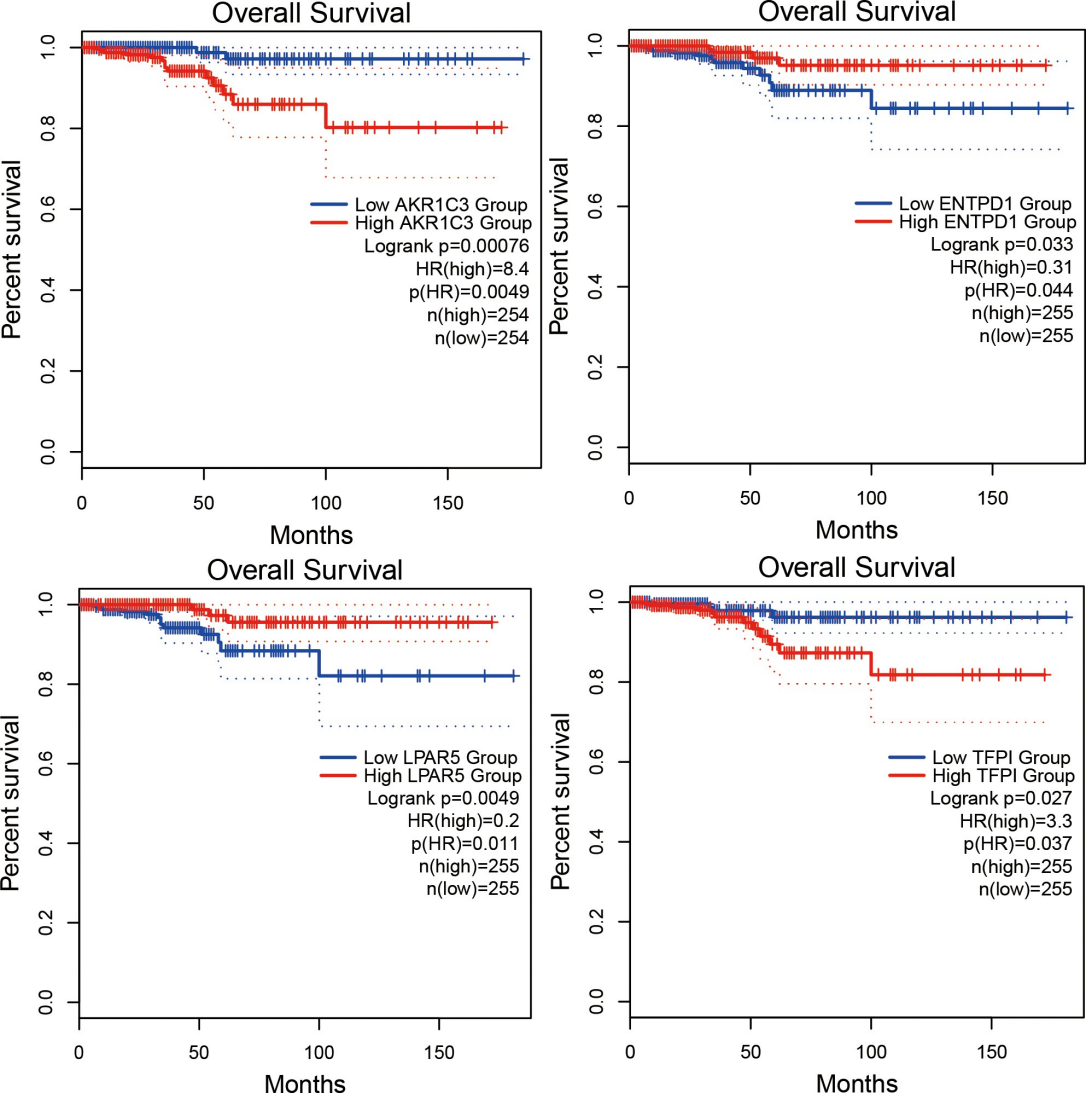
The association between the expression of the four hub genes and the overall survival of patients with PTC via GEPIA analysis. Red curve denotes high expression level, while blue curve denotes low expression level.

**Fig 6:**
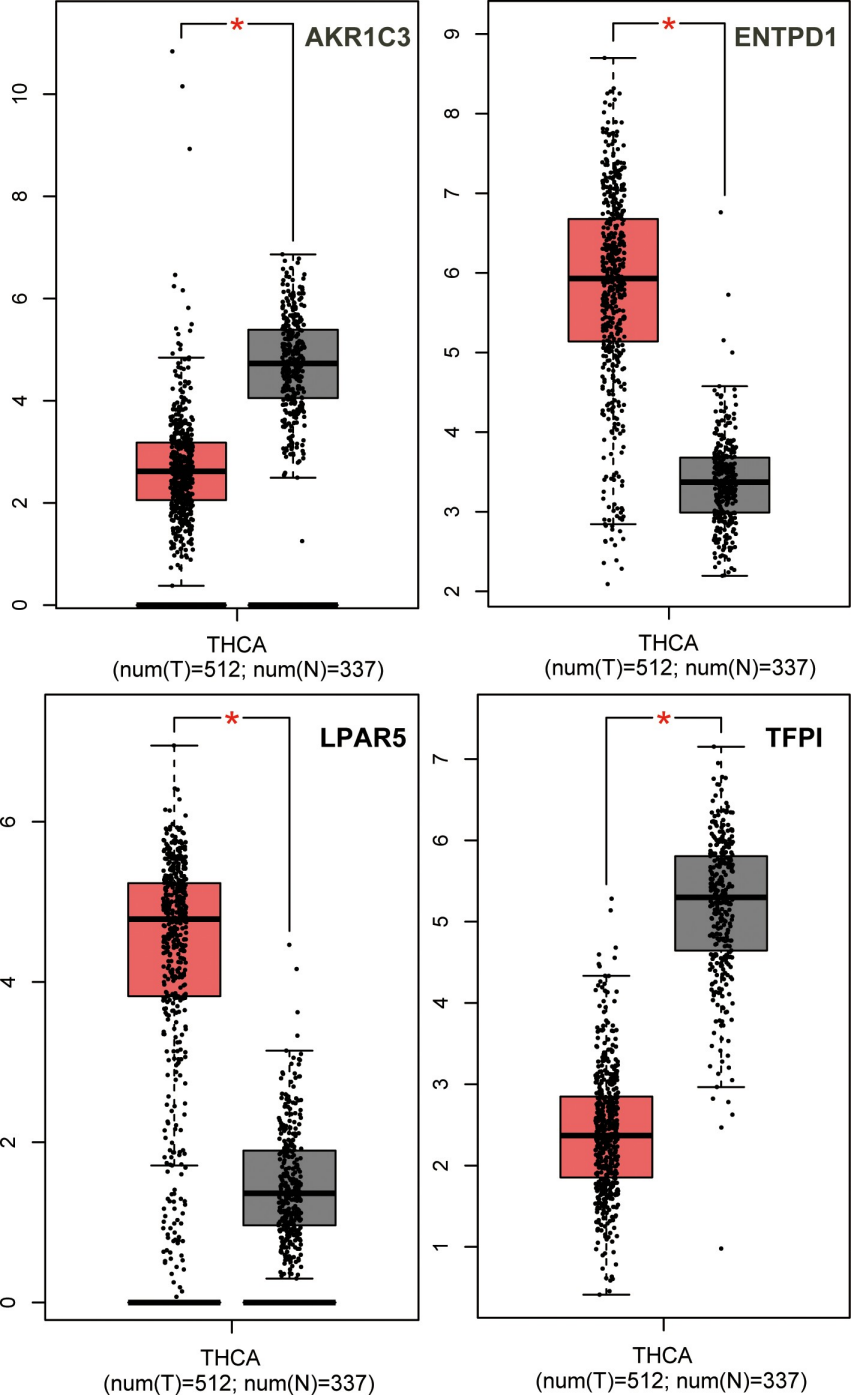
Analysis of hub gene expression levels with GEPIA analysis. The transcript expression comparison of four genes in normal (n = 59) and tumor (n = 505) tissues via GEPIA.

**Fig 7:**
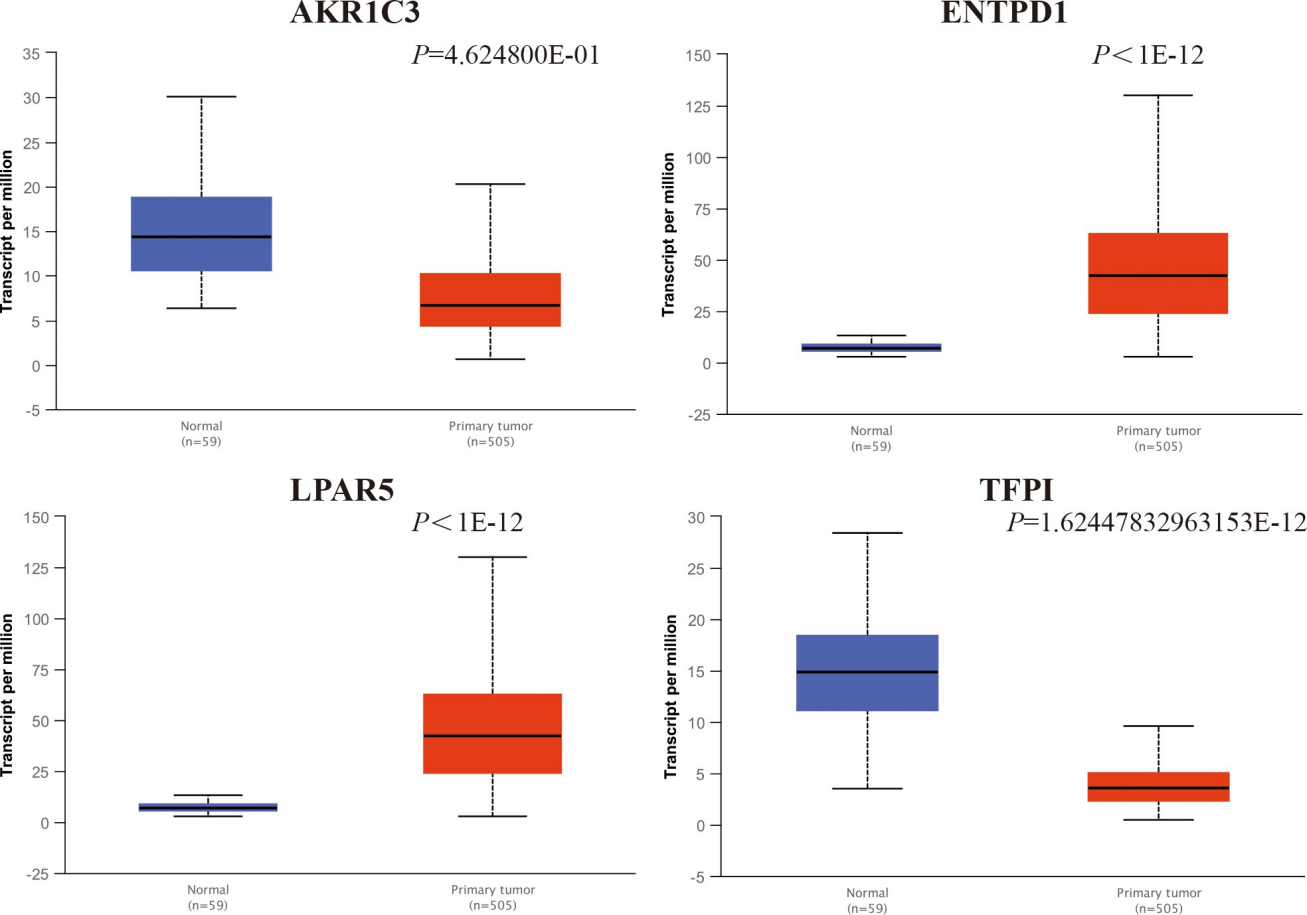
Analysis of hub gene expression levels with UALCAN analysis. The transcript expression comparison of four genes in normal (n = 59) and tumor (n = 505) tissues via UALCAN.

**Fig 8:**
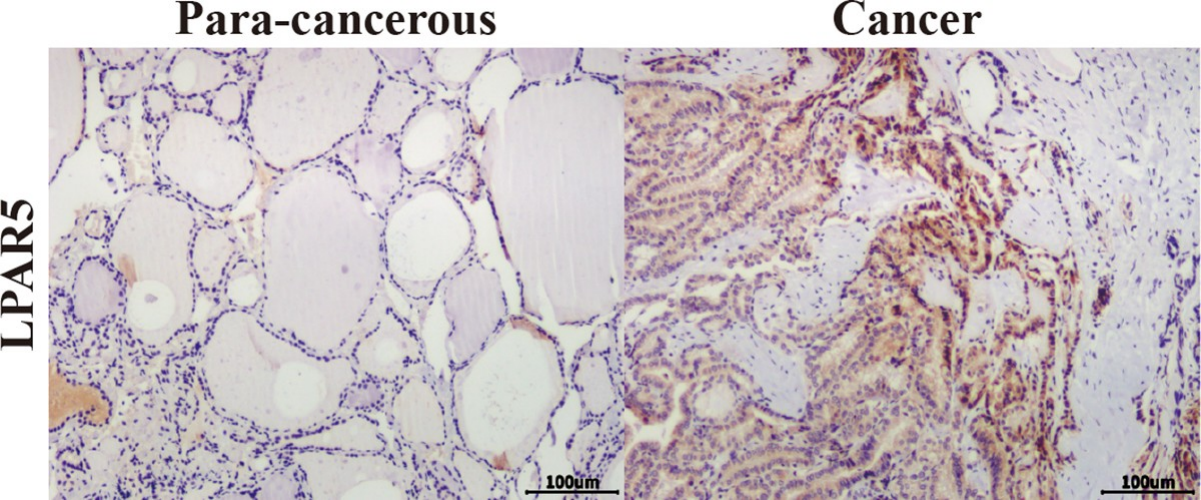
Representative IHC for LPAR5 expression in tissue microarrays (scale bar, 100 μm). The categories of the expression of LAPR5 immunostaining in the papillary thyroid carcinoma at 100× magnification. Left: para-cancerous tissue; right: cross-section of a tumor obtained from PTC patient shows intense cytoplasmic staining.

We used GEPIA to identify the correlation between 30 central genes and the overall survival of patients with PTC. A total of 510 patients with PTC were included in the analysis. Patients with PTC were divided into high- and low-expression groups based on the median values of gene expression. The results suggested that the low expression levels of AKR1C3 (*P* = 0.00076) and TFPI (*P* = 0.027) were significantly correlated with shorter survival of patients with PTC, whereas the high expression levels of ENPD1 (*P* = 0.033) and LPAR5 (*P* = 0.0049) were remarkably related to the short survival of PTC patients (Fig 5). Furthermore, GEPIA was used to validate the expression levels of four hub genes in PTC and normal tissues. The results showed that the expression of all four genes was remarkably different in PTC samples compared to normal samples. ENTPD1 and LPAR5 expression levels were significantly upregulated, while the expression levels of AKR1C3 and TFPI were remarkably downregulated in PTC samples compared to normal samples (Fig 6). Moreover, UALCAN was used to analyze the expression levels of the four hub genes. We found that the expression levels of ENTPD1, LPAR5, and TFPI were remarkably different in PTC tissues compared to normal tissues, except for AKR1C3 (Fig 7). To verify the reliability of the prediction, we selected the LPAR5, TFPI and ENTPD1 protein and analyzed its expression using tissue microarray. The results indicated that LPAR5 expression was remarkably higher in papillary thyroid carcinoma tissues than that observed in the adjacent tissues (Fig 8), while there is no difference in the expression of TFPI and ENTPD1 between PTC tissues and the adjacent tissues (S1 Fig).

## Discussion

In this study, we analyzed gene expression profiles obtained from three GEO datasets (GSE 3678, GSE 33630, and GSE 53157 by GEO2R and identified 136 upregulated and 198 downregulated genes, for a total of 334 DEGs. Particularly, the data sets selected herein were obtained from the same data platform, and the purpose was to ensure data uniformity and reliability. 63 papillary thyroid cancer specimens and 55 normal thyroid specimens were enrolled in this study. Gene Ontology and KEGG pathway enrichment analysis suggested that these DEGs were remarkably enriched in certain pathways, including pathways in cancer, TGFβ receptor signaling pathway, growth factor binding, and were markedly associated with BMP, SMAD, and MAPK signal activity. Thereafter, we constructed a PPI network using the STRING and Cytoscape software and performed screening for 30 vital hub genes from the PPI network complex via MCODE module analysis. Furthermore, we found that 4 of the 30 genes were markedly associated with survival via GEPIA analysis. Expression validation based on GEPIA and UALCAN showed that there was a significant difference in the expression of ENTPD1, LPAR5, and TFPI in PTC and normal tissues, suggesting that these genes might play critical roles in the tumorigenesis and development of PTC.

Previous research has indicated that the three genes identified in our study are involved in the initiation and development of tumors. Lysophosphatidic acid (LPA) is a biologically active mediator that affects cellular functions, such as regulation of cell proliferation, transcellular migration, differentiation, morphogenesis, and prevention of apoptosis [14, 15]. LPAR5 is a member of the G protein-coupled transmembrane receptor that establishes interaction with LPA [16, 17]. The cells subjected to treatment with 12-O-tetradecanoylphorbol-13acetate (TPA) showed higher motility than control cells, whereas LPAR5 knockdown reversed this phenomenon [18]. Recent studies on PTC have shown that LPAR5 is associated with progression and overall survival in thyroid cancer, which is consistent the findings presented in our study [19–21]. Tissue factor pathway inhibitor (TFPI) is an endogenous inhibitor of tissue factor-induced blood coagulation, and its expression has been demonstrated in smooth muscle cells, monocytes, platelets, and several breast cancer cell lines [22–24]. Wang et al. suggested that downregulation of TFPI might result in tumor cell growth and migration, and the suppression of TFPI by hypoxia microenvironment might be one of the supervisory mechanisms by which hypoxia could promote angiogenesis and tumor growth [25]. miR-500 inhibition could suppress the proliferation and invasion of prostate cancer cells and tumorigenicity in vivo, while TFPI knockdown reversed these effects [26]. The expression level of TFPI in luminal-A breast cancer patients was significantly lower than that in healthy volunteers [27]. Zarychta et al. claimed that TF seemed to be a tumor-promoting factor, while TFPI exhibited tumor suppressor properties [28]. Erem et al. found a significant decrease in TFPI levels in patients with hyperthyroidism [29]. ENTPD1, which encodes the protein CD39, is extremely important for the production of immunosuppressive adenosine [30]. Mosaad Zaki et al. confirmed that the expression level of ENTPD1 on CD4+ T helper cells in chronic lymphocytic leukemia (CLL) patients was significantly higher than that in the controls, and ENTPD1 and CD4 expression levels were remarkably expressed in high-risk CLL patients [31]. Cai et al. found that ENTPD1 overexpression was a predictor of poor prognosis in GC patients after subjection to radical gastric cancer (GC) resection [32]. Pathological research has shown that the expression of ENTPD1 in head and neck squamous cell carcinoma (HNSCC) is positively correlated with tumor stage, which may predict a poor prognosis [33]. Interestingly, Sun et al. found that ENTPD1 deletion promoted the development of both induced and spontaneous autochthonous liver cancer in mice [34]. These results suggest that the development of immunotherapy targeting CD39 may be promising. Thus far, there have been a few reports on the relationship between ENTPD1 and thyroid cancer. Thus, the specific functions of ENTPD1 in thyroid cancer should be further explored. However, further experiments are warranted to explore the pathogenesis and molecular mechanisms of the hub genes in PTC.

## Conclusions

In summary, our integrated bioinformatics study presented three hub genes LPAR5, and pathways associated with PTC which might be a reliable as potential biomarkers, and might provide new insights into the diagnosis, prognosis, and target therapy for PTC.

## Supporting information

S1 Fig (TIF)

## Abbreviations

PTC: Papillary thyroid cancer
GEO: Gene Expression Omnibus
DEGs: Differentially expressed genes
DAVID: Database for Annotation, Visualization, and Integrated Discovery
GO: Gene ontology
KEGG: Kyoto Encyclopedia of Gene and Genome
BP: Biological process
CC: Cellular component
MF: Molecular function
STRING: Search Tool for the Retrieval of Interacting Genes/Proteins
MCODE: Molecular Complex Detection
GEPIA: Gene Expression Profiling Interactive Analysis
TCGA: The Cancer Genome Atlas
AKR1C3: Aldo-keto reductase family 1 member C3
LPAR5: Lysophosphatidic acid receptor 5
TFPI: Tissue factor pathway inhibitor
ENTPD1: Ectonucleoside triphosphate diphosphohydrolase 1
MAPK: Mitogen-activated protein kinase
BMP: Bone morphogenetic protein
TPA: 12-O-tetradecanoylphorbol-13acetate
CLL: chronic lymphocytic leukemia
GC: gastric cancer
HNSCC: squamous cell carcinoma of the head and neck
