# Supplementary figures and images for: Integrated bioinformatics analysis and screening of hub genes in papillary thyroid carcinoma
Source: PLoS One. 2021 Jun 11;16(6):e0251962. doi: 10.1371/journal.pone.0251962 (PMC8195368; doi:10.1371/journal.pone.0251962)

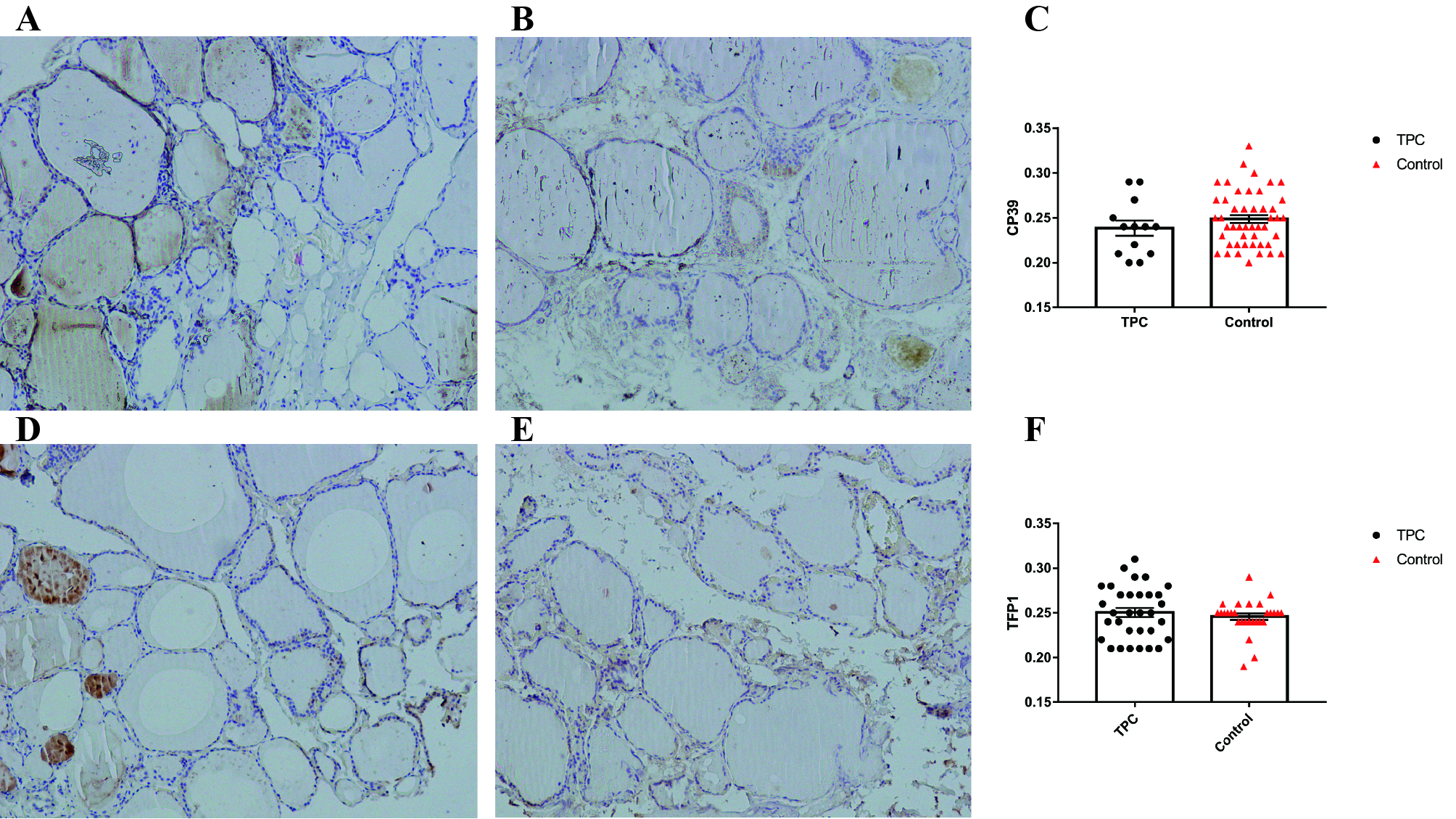

Supplement: S1 Fig — (TIF) [file pone.0251962.s001.tif]
